# Knowledge, attitudes and practices of critical care unit personnel regarding pediatric palliative care: a cross-sectional study

**DOI:** 10.1186/s12904-024-01456-w

**Published:** 2024-05-21

**Authors:** Hua Lu, Linfei Jin

**Affiliations:** Department of Peadiatric Intensive Care Unit, Shanghai Children’s Medical Center, School of Medicine, Shanghai Jiao Tong University, Shanghai, 200127 China

**Keywords:** Palliative Care, Palliative Medicine, Pediatrics, Pediatric Intensive Care Units, Surveys and questionnaires, Knowledge, attitudes, Practic

## Abstract

**Background:**

Few studies have evaluated the perceptions of healthcare providers in China regarding pediatric palliative care, particularly in critical care units (PICUs), where many children receive palliative care. To evaluate the knowledge, attitudes and practices of PICU personnel in China regarding pediatric palliative care.

**Methods:**

This cross-sectional study was conducted in five cities in China (Shanghai, Suzhou, Chongqing, Chengdu and Yunnan) between November 2022 and December 2022.

**Results:**

The analysis included 204 participants (122 females), with 158 nurses and 46 physicians. The average knowledge, attitude and practice scores were 9.75 ± 2.90 points (possible range, 0–13 points), 38.30 ± 3.80 points (possible range, 12–60 points) and 35.48 ± 5.72 points (possible range, 9–45 points), respectively. Knowledge score was higher for physicians than for nurses (*P* < 0.001) and for personnel with previous training in pediatric palliative care (*P* = 0.005). According to structural equation modelling knowledge had a direct positive effect on attitude (β = 0.69 [0.28–1.10], *p* = 0.001), and indirect on practice (β = 0.82 [0.36–1.28], *p* < 0.001); attitude had significant effect on practice as well (β = 1.18 [0.81–1.56], *p* < 0.001).

**Conclusions:**

There is room for improvement in the knowledge, attitudes and practices of PICU personnel in China regarding pediatric palliative care. The findings of this study may facilitate the design and implementation of targeted education/training programs to better inform physicians and nurses in China about pediatric palliative care.

**Supplementary Information:**

The online version contains supplementary material available at 10.1186/s12904-024-01456-w.

## Background

Many children worldwide suffer from life-limiting conditions (from which they will die because there is no reasonable hope of cure) or life-threatening conditions (from which they will die if potentially curative treatment fails) [1]. Life-limiting and life-threatening conditions encompass a wide range of illnesses including cancers, nervous system diseases, congenital anomalies, infectious diseases, and conditions affecting the circulatory and respiratory systems [2]. The prevalence of life-limiting conditions in the pediatric population has increased in recent years and is predicted to continue to increase during the next decade [3]. Estimates of the prevalence of life-limiting conditions in children and young people range from 43.2 per 10,000 to 95.5 per 10,000 [3–5].

Pediatric palliative care involves the provision of physical, psychological and spiritual care to children with life-threatening illnesses and support to their family members so as to optimize the quality of life of the children and their families [6]. It has been estimated that more than 21 million children worldwide require palliative care each year, with requirements varying between different countries [7]. In China, it has been estimated that more than three million children require palliative care [7]. Pediatric palliative care is provided by a multidisciplinary team that includes doctors, nurses, social workers, psychologists and therapists, and a high level of care for the dying patient requires excellent communication and shared decision making between medical personnel, patients and their families [6]. International standards for pediatric palliative care have been published [8], and the provision of specialized pediatric palliative care has been reported to enhance patient quality of life [9] and reduce healthcare system costs [10]. However, there are numerous barriers to the implementation of effective pediatric palliative care including family-related factors [11], misperceptions and lack of knowledge among healthcare providers [12, 13], insufficient funding, and inadequate organization and integration with other pediatric services [14, 15].

Although pediatric palliative care services are improving in China, it is widely recognized that advances need to be made more rapidly. The Chinese Government published practice guidelines for hospice care in 2017 [16] and selected pilot regions for the implementation of hospice services. In 2019, 71 areas were selected for the promotion of pediatric palliative care [17]. Nevertheless, specialized pediatric palliative care services remain limited in many cities in China due to a shortage of resources, and in many children’s hospitals, palliative care beds are provided by hematology or oncology departments rather than specialized centers. The Butterfly House at Changsha First Social Welfare Institute was the first hospice center for children to be built in China, and Daisy House in Beijing Songtang Hospital is the only family-type pediatric palliative care center in China. In addition to the limited number of specialized centers, it is recognized that there are various barriers to palliative care among medical personnel in China [18, 19]. However, few studies have evaluated the perceptions of healthcare providers in China regarding pediatric palliative care, particularly in critical care units (PICUs), where many children receive palliative care [20].

Knowledge, attitude and practice (KAP) surveys provide important information concerning the knowledge, attitudes, beliefs, misconceptions and behaviors of medical professionals towards a health-related topic, both at the baseline and as a tool to assess the efficacy of educational intervention [21, 22]. A few previous studies utilized the KAP method to successfully guide the development and implementation of training programs for healthcare personnel in palliative care, reporting promising results [22, 23]. Therefore, the aim of this study was to evaluate the knowledge, attitudes and practices of PICU personnel in China with regard to pediatric palliative care.

## Methods

### Study design and participants

This cross-sectional, questionnaire-based study enrolled medical personnel working in PICU s in five cities in China between November 2022 and December 2022. The inclusion criteria were: (1) physicians or nurses working in a PICU in Shanghai, Suzhou, Chongqing, Chengdu or Yunnan; (2) at least one year of work experience; and (3) volunteered to participate in this study. Medical personnel engaged in other studies, training programs or internships were excluded. This study was approved by the ethics committee of Shanghai Children’s Medical Center affiliated to Shanghai Jiao Tong University School of Medicine (SCMCIRB-K2022169-1), and all participants provided informed written consent.

### Questionnaire design and distribution

The first draft of the questionnaire was designed with reference to the “2022 Society of Critical Care Medicine Clinical Practice Guidelines on Prevention and Management of Pain, Agitation, Neuromuscular Blockade, and Delirium in Critically Ill Pediatric Patients With Consideration of the ICU Environment and Early Mobility”, regarding analgesia, sedation, neuromuscular blockade, delirium, iatrogenic withdrawal, end-of-life care and PICU environment optimization [24]. The questionnaire was then modified according to the comments of five medical professionals with expertise in pediatric palliative care (four clinicians and one social worker). According to their input, all sections of questionnaire were revised, adding types of received training, knowledge of relationship between palliative therapy and primary positive treatment, views on the integration of critical care medicine/nursing into palliative therapy and withdrawal support devices. A pilot study was performed by distributing the questionnaire to 57 medical personnel, and the Cronbach’s α coefficient of the questionnaire was determined to be 0.935, which suggested excellent internal consistency (i.e., excellent reliability).

The final version of the questionnaire was in Chinese and consisted of 43 questions across four dimensions: demographic information, knowledge, attitude and practice. The demographic information dimension consisted of 9 items that collected the following data: age, gender, occupation (physician or nurse), education level, years of professional work experience, location of the hospital at which employed, previous training in pediatric palliative care, type of training received, and whether pediatric palliative care was available in their department. The knowledge dimension contained 13 items (K1–K13), each of which was scored 1 point for a correct answer and 0 points for an incorrect or unclear answer. The total score of the knowledge dimension ranged from 0 to 13 points. The attitude dimension comprised 12 items (A1–12). The possible responses for items A1–A7 were “strongly agree”, “agree”, “neutral”, “disagree” and “strongly disagree”, while those for items A8–A12 were “very greatly”, “greatly”, “moderately”, “slightly” and “very slightly”. Each item in the attitude dimension was scored using a 5-point Likert scale (1–5 points) according to the positivity/negativity of the response selected. Each of items A8–A12 was divided into three parts, and the average of the scores for the three parts was used as the item score. The total score of the attitude dimension ranged from 12 to 60 points. The practice dimension comprised 9 items (P1–P9), with item P9 divided into two parts (the score for item P9 was calculated as the average of the scores for the two parts). The items were scored using a 5-point Likert scale (“always” = 5 points, “often” = 4 points, “sometimes” = 3 points, “rarely” = 2 points, and “never” = 1 point). The total score for the practice dimension ranged from 9 to 45 points.

An online questionnaire was constructed using an online survey tool (SoJump), and a QR code that linked to the online questionnaire was distributed to the participants via WeChat. To ensure the quality and completeness of the questionnaire results, each IP address could only be used once for submission, and all items in the questionnaire had to be completed before submission was permitted. All questionnaires were checked for completeness, consistency and validity by members of the research team.

### Statistical analysis

Based on international principles of questionnaire design and previous research experience, it is generally recommended that the sample size should be 5 to 10 times the number of questionnaire items [25]. With 34 KAP items of the questionnaire, the required sample size was 170. SPSS 26.0 (IBM Corp., Armonk, NY, USA) was used for the analyses. Quantitative data were tested for normality by the Shapiro-Wilk’s test. To ensure consistency in data presentation, all variables were expressed as mean ± standard deviation (SD). Variables that met the normal distribution were compared between groups using the t-test or one-way analysis of variance (ANOVA; three or more groups), while non-normally distributed were compared between groups using the Mann-Whitney U test or Kruskal-Wallis test. Categorical data are expressed as frequency (percentage) and were analyzed using the chi-squared test.

Structural equation modelling (SEM) was used to test the initial hypotheses that knowledge regarding pediatric palliative care has effect on attitude, and attitude has effect on practice. A two-sided *P* < 0.05 was considered statistically significant.

## Results

### Demographic characteristics

A total of 204 medical professionals (175 females, 85.78%), including 158 nurses (77.45%) and 46 physicians (22.55%), participated in the survey. The demographic characteristics of the study participants are shown in Table S1. Just over half the respondents (111/204, 54.41%) were aged ≥ 30 years-old, and only a minority of the participants (38/204, 18.63%) had a master’s degree or higher. Around one-third of the respondents (70/204, 34.31%) had ≤ 5 years of professional experience. Only a minority of participants had received training in pediatric palliative care (147/204, 72.06%), which was mainly theory-based (53/204, 25.98%). Approximately half of the PICU personnel (97/204, 47.55%) indicated that pediatric palliative care was available in their department.

### Knowledge scores

The average knowledge score was 9.75 ± 2.90 points (possible range, 0–13 points), indicating that the respondents had a moderate level of knowledge about pediatric palliative care. The distribution of the responses to each of the 13 questions in the knowledge dimension are shown in Table S2. More than 85% of the respondents correctly answered questions regarding the aims of pediatric palliative care (86.27%; item K1), the importance of controlling pain/other symptoms, ensuring patient comfort and supporting parents/caregivers (90.20%; item K4), the use of pain scales to assess pain (94.61%; item K6), the importance of parents/caregivers being present during routine care (87.25%; item K9), sleep deprivation being a major stressor in patients with life-limiting conditions (91.67%; item K10), the importance of incorporating pediatric palliative care from outset following diagnosis (86.76%; item K11), and the role of pediatric palliative care in quality of life improvement (85.29%; item K12). However, nearly half of the respondents incorrectly believed that opioid substitution therapy should be considered to reduce iatrogenic withdrawal syndrome regardless of the previous dose used, duration of therapy or drug utilized (46.57%; item K7).

As shown in Table S1, subgroup analyses revealed that the knowledge score was higher for physicians than for nurses (*P* < 0.001) and for personnel who had received previous training in pediatric palliative care (*P* = 0.005).

### Attitude scores

**Fig. 1:**
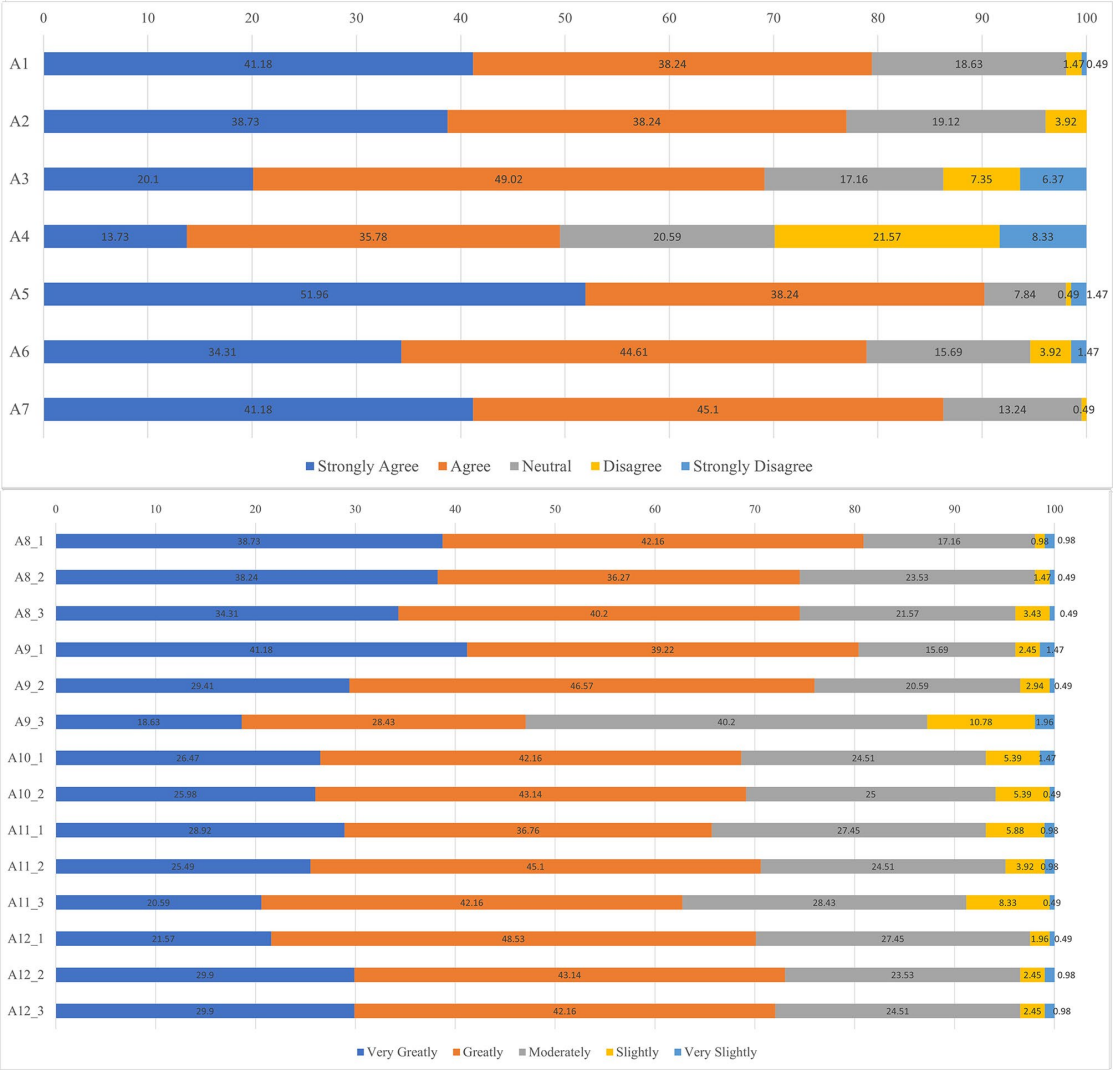
Distribution of responses to the questions in the attitude dimension. (**A**) Items A1–A7. A1: Pediatric palliative care should be implemented to optimize quality of life if invasive therapy would cause discomfort and have little effect on the underlying disease (positive). A2: Pediatric palliative care can improve outcomes for children and their families (positive). A3: Pediatric palliative care can make the family lose hope (negative). A4: Pediatric palliative care will result in the family being placed under pressure in a variety of ways (negative). A5: Pediatric palliative care should be implemented by a multidisciplinary team composed of doctors, nurses, social workers, pharmacists, physiotherapists and others (positive). A6: Pediatric palliative care should be integrated into the intensive care unit and jointly provided by the Department of Critical Care Medicine and palliative care team (positive). A7: Nurses, educators and researchers should do more to provide guidance for the application and evaluation of high-quality pediatric palliative care (positive). **(B)** Items A8–A12. Which of the following factors influence pediatric palliative care: A8: political-economic factors (negative); A8.1: insufficient human resources and inadequate structural organization; A8.2: insufficient financial resources and drug availability; A8.3: level of medical insurance reimbursement. A9: medical personnel-related factors (negative); A9.1: Time pressures; A9.2: lack of education/training/knowledge (communication skills, pain assessment and management); A9.3: emotional distress/discomfort/sadness. A10: family-related factors (negative); A10.1: misunderstanding of the role of palliative care and fear of being abandoned by medical personnel; A10.2: misunderstanding of the prognosis or goals of treatment. A11: social factors (negative); A11.1: views that promote life and are not compatible with treatment withdrawal; A11.2: misunderstanding of pediatric palliative care (the role of the hospice, voluntary abandonment); A11.3: treatment/cure-oriented views. A12: clinical implementation and standardization-related factors (negative); A12.1: timing of the initiation of palliative care and uncertainty about the prognosis; A12.2: lack of specific reference guidelines; A12.3: lack of specific implementation standards (team formation, bereavement care, etc.)

The average attitude score was 38.30 ± 3.80 points (possible range, 12–60 points), implying that the surveyed anesthetists did not have a strongly positive attitude to pediatric palliative care. The distributions of the responses to the 13 questions in the attitude dimension are summarized in Fig. 1. The vast majority of respondents gave very positive or positive responses to questions regarding the selection of palliative care to maintain the patient’s quality of life when invasive therapy might cause discomfort and have little effect on the underlying disease (79.42%; item A1), the role of palliative care in improving outcomes for patients and families (76.97%; item A2), the influence of palliative care on the hopes of the family (69.12%; item A3), the multidisciplinary nature of a palliative care team (90.20%; item A5), the importance of integrating pediatric critical care into the PICU (78.92%; item A6), and the need for education, training and evaluation to ensure high-quality pediatric palliative care (86.28%; item A7). However, a mixed distribution of responses was obtained for the question asking whether palliative care would lead to the patient’s family being put under pressure in a variety of ways (item A4). More than 80% of the respondents considered that pediatric palliative care was influenced by insufficient human resources/inadequate organization (80.89%; item A8.1), time pressures at work (80.40%; item A9.1) and a lack of education/training/knowledge (80.40%; item A9.2).

The vast majority of demographic characteristics had no significant influence on the attitude score (Table S1). The only exception was technology-based training, which was associated with a slightly higher attitude score (*P* = 0.004). A more detailed analysis of the attitude dimension scores revealed that physicians had significantly higher scores than nurses for items A1–A4 (considerations for the selection of pediatric palliative care; *P* = 0.026) and items A5–A7 (composition of the palliative care team; *P* = 0.023), whereas the scores were similar for items A8–A12 (Table S3).

### Practice scores

**Fig. 2:**
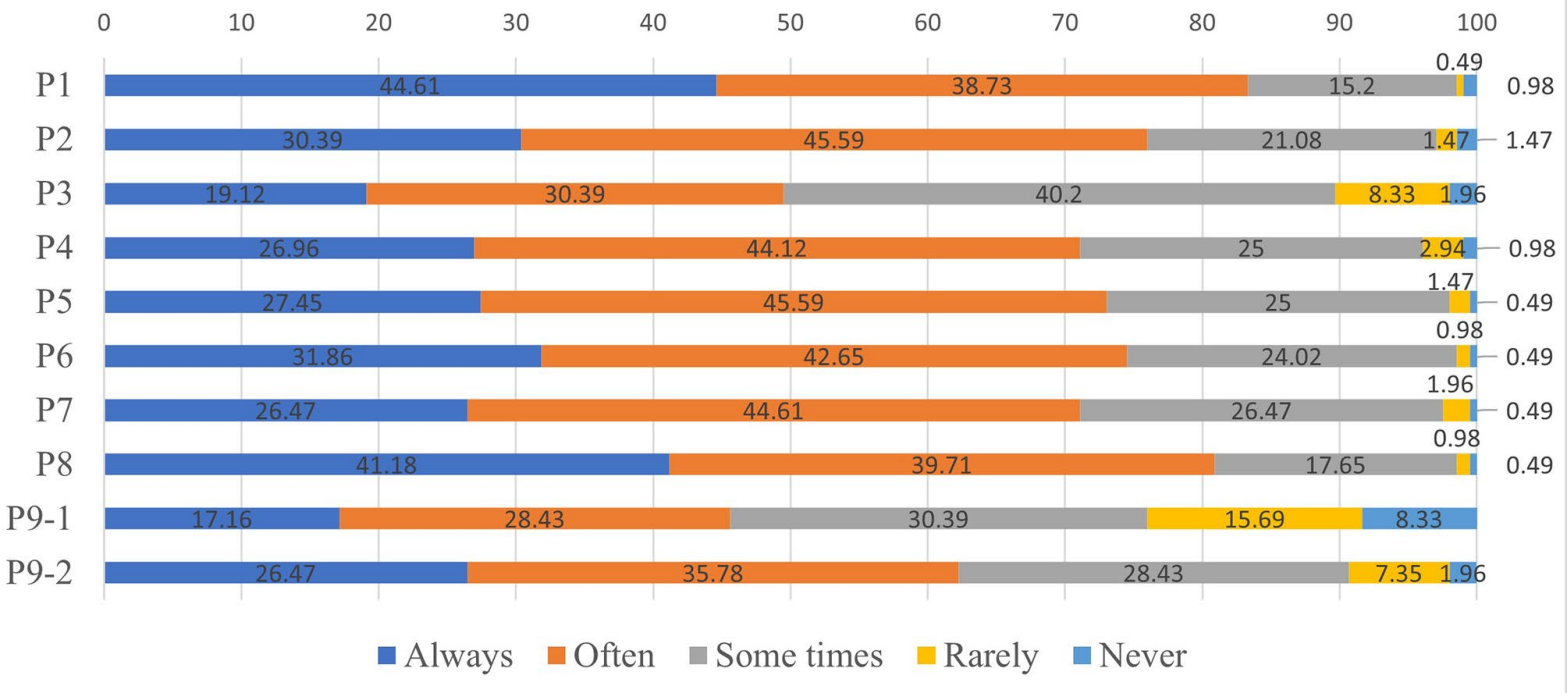
Distribution of responses to the questions in the practice dimension. How frequently do you (and your team) implement the following if necessary. P1: analgesia (positive); P2: sedation (positive); P3. application of neuromuscular blockers (positive); P4: screening and prevention of delirium (e.g., minimizing overall sedative exposure) (positive); P5: assessment, prevention and mitigation of iatrogenic withdrawal syndrome (positive); P6: environment optimization (positive); P7: basic symptom management (positive); P8: high-quality communication (positive); P9: end-of-life care (positive); P9.1: withdrawal of life support equipment; P9.2: support for parents

The practice score for the respondents averaged 35.48 ± 5.72 points (possible range, 9–45 points), suggesting that there was room for improvement in the practices of the PICU personnel. More than 80% of participants indicated that they and their team always or often implemented analgesia (83.34%; item P1), screening and interventions to prevent delirium (83.34%; item P4), and high-quality communication (80.89%; item P8) when needed (Fig. 2).

The practice scores were comparable between subgroups stratified according to the various demographic characteristics (Table S1). Further analysis indicated that nurses had a significantly higher score than physicians for item P3 (application of neuromuscular blockers; *P* < 0.001), whereas physicians had a higher score for item P9 (end-of-life care; *P* < 0.001; Table S3). However, there were no significant differences in the other item scores between physicians and nurses, and training was without significant effect on the individual practice item scores (Table S3).

### SEM

**Fig. 3:**
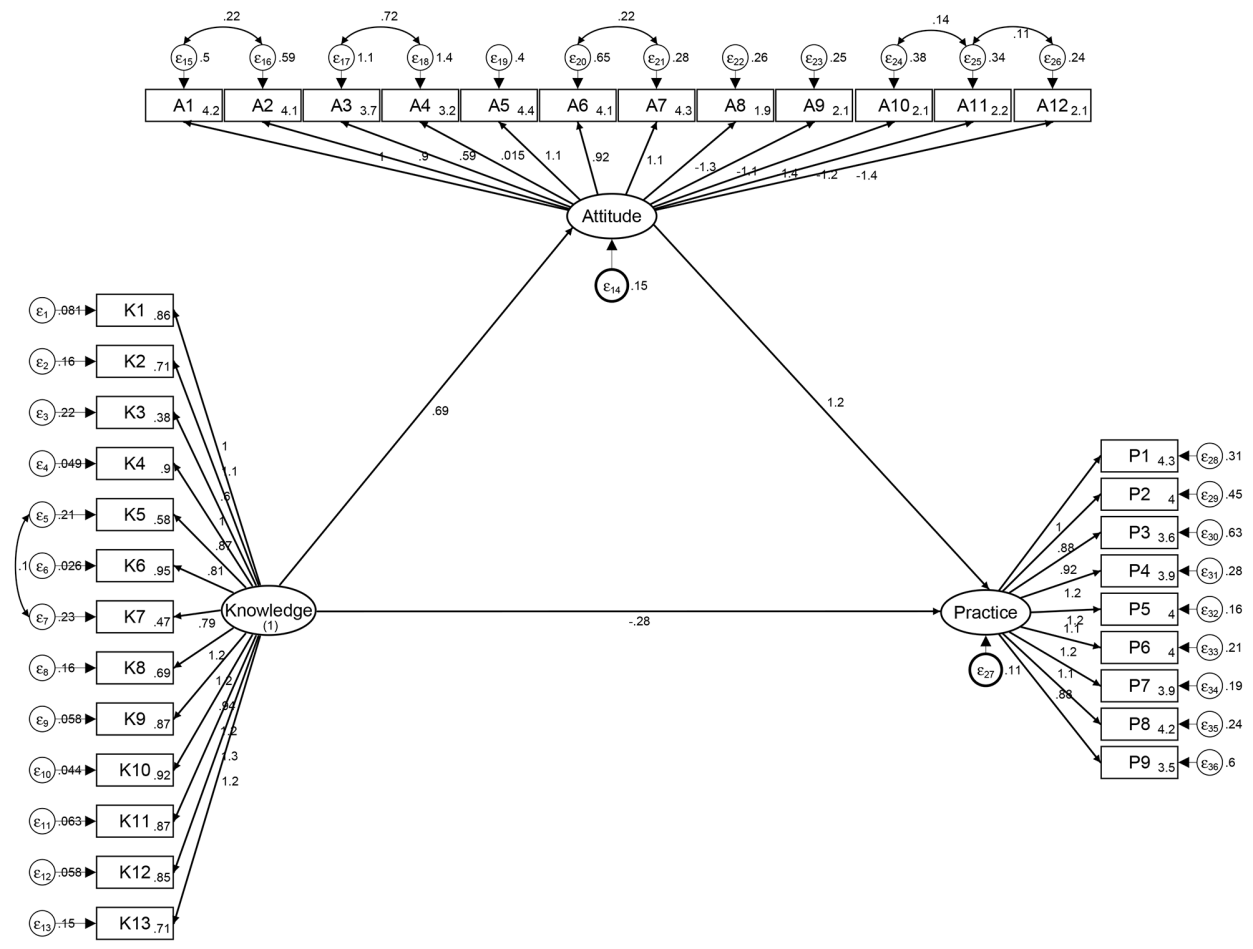
Structural equation model of KAP. K: knowledge; A: attitude; P: practice (*denotes statistical significance: *p* < 0.05)

Method of SEM was used to explore the factors that might influence KAP scores, with fit indices demonstrating acceptable model fit (Fig. 3 and Table S4). It was found that knowledge had a direct positive effect on attitude (β = 0.69 [0.28–1.10], *p* = 0.001), as well as significant indirect (β = 0.82 [0.36–1.28], *p* < 0.001) effect on practice. The effect of attitude on practice was significant as well (β = 1.18 [0.81–1.56], *p* < 0.001) (Table S5).

## Discussion

It was found that the knowledge score was higher for physicians than for nurses and for personnel with previous training or greater professional experience. Demographic characteristics had only limited effects on the attitude and practice scores, while knowledge had a direct positive effect on attitude and indirect on practice. To our knowledge, this is the first survey evaluating the knowledge, attitudes and practices of PICU personnel regarding pediatric palliative care. This study provides new insights into the knowledge, attitudes and practices of physicians and nurses in Chinese PICUs with regard to pediatric palliative care. The results of this study may be used for designing and development of targeted education and training interventions to support PICU personnel providing pediatric palliative care.

### Knowledge gaps identified after analysis of study results

In the present study, 7 of the 13 questions in the knowledge dimension were answered correctly by more than 85% of the surveyed PICU personnel, including items relating to the aims of pediatric palliative care (item K1), the importance of controlling pain/symptoms to make the patient comfortable and supporting parents/caregivers (item K4), the use of pain scales to assess pain (item K6), the importance of parents/caregivers being present during routine care (item K9), sleep deprivation as a major stressor in patients receiving palliative care (item K10), the importance of the early initiation of pediatric palliative care after diagnosis (item K11), and the benefits of pediatric palliative care on patient quality of life (item K12). In addition, more than two-thirds of the participants were aware that pediatric palliative care is provided for a wide range of non-malignant as well as malignant conditions (item K2), that patient comfort is an important aim, with unnecessary examinations and treatments avoided (item K8), and that active treatment to prolong survival should not be promoted if it compromises quality of life (item K13). However, nearly 40% of the medical personnel incorrectly believed that pediatric palliative care is only given to patients with life-threatening diseases after treatment has failed (item K3), and nearly half of the respondents incorrectly believed that opioid substitution therapy should be considered to reduce iatrogenic withdrawal syndrome regardless of the dose used, the duration of therapy or the drug utilized (item K7). Notably, the average knowledge score was 38.30 ± 3.80 points out of a possible maximum of 60 points. The above findings highlight knowledge gaps among the surveyed physicians and nurses, particularly with regard to the indications for palliative care and opioid use in children receiving palliative care.

### Occupational, but not demographic factors influenced knowledge scores

The knowledge levels of the participants in the present study are broadly comparable to those reported previously by surveys of clinicians and nurses. For example, Zuniga-Villanueva et al. described a mean score of 6.8 out of 10 for pediatricians in Mexico [26]. Abuhammad et al. found that nurses in Jordan had a low score in knowledge of pediatric palliative care [27]. Zeru et al. determined that only 62.8% of surveyed nurses in Ethiopia had a good level of knowledge [28]. Similarly, Ghoshal et al. reported that less than half of the surveyed doctors and nurses in India had a good level of knowledge (defined as a knowledge score ≥ 70%) [22]. Detsyk et al. found that 25.3% of healthcare workers providing medical services to children (including nurses, general practitioners and pediatricians) did not know the meaning of pediatric palliative care, with 71.5% of the respondents believing it was mainly provided to patients with cancer, and only 54.8% of the respondents being aware that it was provided to children with incurable chronic diseases [29]. Additionally, only 59.7% of the respondents in the study of Detsyk et al. knew that palliative care should be initiated at the time of diagnosis of an incurable disease, while only 52.6% of the participants were aware that palliative care should also offer support to the relatives of seriously ill children [29]. In agreement with our data suggesting a deficiency in knowledge regarding opioid use, Stenekes et al. concluded that healthcare providers in Canada had knowledge gaps related to opioid use and the development of tolerance to opioids and sedatives [30]. Madden et al. also found variation in the level of comfort with different opioids among physicians in the USA [31].

In this study the subgroup analyses demonstrated that the knowledge score was higher for physicians than for nurses. Furthermore, a higher knowledge score was associated with previous training and working in a department where pediatric palliative care was available. Our findings are consistent with prior research concluding that a higher level of knowledge in pediatric palliative care was associated with training and greater experience in palliative care [26–28, 32, 33]. Occupation type has also been reported to influence knowledge level [29]. We suggest that the implementation of education and training programs, such as those described previously [22, 27, 32, 33], may help to improve the knowledge of PICU personnel in China regarding pediatric palliative care.

### Specific believes formed the mostly positive attitude

The mean attitude score of 38.30 ± 3.80 points out of maximum 60 points indicates that, overall, the participants in this study had a moderately positive attitude toward pediatric palliative care. The majority of respondents (> 69%) gave “very positive” or “positive” answers to 6 of the first 7 questions in the attitude dimension (A1–A3, A5–A7), whereas the responses for item A4 varied. More than 60% of the respondents believed that pediatric palliative care was influenced by economic (item A8), personnel-related (item A9), family-related (item A10), social (item A11) and implementation-related (item A12) factors. In addition, structural equation modelling confirmed that knowledge had a direct positive effect on attitude, although influence of knowledge or attitude on practice was not significant. Previous studies have also reported a variety of factors believed to affect the implementation of effective pediatric palliative care, including family preference for life-sustaining treatment, family not ready to acknowledge an incurable condition, parent discomfort with the possibility of hastening death [11], inadequate training [12], clinician misperceptions and emotional burden, prognostic uncertainty about treatment options [13], socio-cultural factors, nature of the patient and disease, insufficient training, regulatory/political issues [14], lack of adequate funding, lack of palliative care programs, difficulty integrating palliative care into existing pediatric care at the organizational level, and lack of knowledge [15].

### New education and training programs are needed to further improve practice

The average practice score was 35.48 ± 5.72 points (possible range, 9–45 points), which suggests that there was room for improvement in the practices of the PICU personnel enrolled in this study. Most respondents (> 71%) indicated that, when required, they and their team always/often took care of analgesia, sedation, environment optimization, symptom management and communication, as well as screening and interventions to prevent delirium, or mitigate against iatrogenic withdrawal syndrome. However, only around half of the participants reported to have experience using neuromuscular blockers when indicated. A previous study in Holland reported that neuromuscular blockers were administered in 16% of cases at the time of withholding/withdrawing life-sustaining treatment [34]. Less than half of the respondents in the present study stated that they or their team withdrew life-sustaining treatment when necessary, and less than two-thirds provided support to the parents/caregivers of patients receiving end-of-life care. Overall, our findings concur with those of other studies reporting suboptimal practices [22, 35, 36].

Somewhat unexpectedly, despite being associated with higher knowledge scores, previous training appeared to have little or no effect on the attitudes and practices of PICU personnel regarding pediatric palliative care; practice was influenced by knowledge mostly indirectly, via attitude. This emphasizes the need for improved education and training programs that better target suboptimal attitudes and practices. In line with previous studies in various settings, some crucial aspects for the implementation of a pediatric palliative care program could be supported by the KAP approach, in particular collecting data to characterize the need for pediatric palliative care [22, 23, 37]. Additionally, KAP assessment would help to raise awareness among hospital administration and clinical personnel about pediatric palliative care, and potentially aid in forming a balanced multidisciplinary palliative care team. Thus, above results may be used for the providing education and training to clinical personnel to support continuous provision of pediatric palliative care in PICU.

### Strengths and limitations of the study

This multicenter study used KAP methodology and validated questionnaire to address the issue of the availability and barriers in implementation of pediatric palliative care; the results were additionally strengthened by application of SEM model which allowed to explore the potential influence of other factors on practice scores. Study population included a wide variety of ICU professionals, with different age and experience; still the sample size may be small to detect some specific differences between groups. Participants from five big cities in China answered the questionnaire, allowing to increase the generalizability of the results, but local peculiarities should still be taken into account. Although the KAP questionnaire was developed based on published recommendations and demonstrated good reliability, it may have limitations with regard to its ability to assess perceptions of pediatric palliative care. Finally, this study did not evaluate whether education/training programs would enhance the questionnaire scores, which is of interest for the future research.

## Conclusion

The findings of this study provide new insights into the knowledge, attitudes and practices of PICU personnel in China regarding pediatric palliative care. We anticipate that the results may help guide the development and implementation of education and training programs to improve the implementation of pediatric palliative care by PICU personnel in China.

### Electronic supplementary material

Below is the link to the electronic supplementary material.

Supplementary Material 1

Supplementary Material 2

## Data Availability

All data generated or analysed during this study are included in this published article [and its supplementary information files].

## Abbreviations

PICUs: Particularly in critical care units
KAP: Knowledge, attitude and practice
SEM: Structural equation modelling
