# Supplementary material for: Knowledge, attitudes and practices of critical care unit personnel regarding pediatric palliative care: a cross-sectional study
Source: BMC Palliat Care. 2024 May 21;23:125. doi: 10.1186/s12904-024-01456-w (PMC11106871; doi:10.1186/s12904-024-01456-w)
Supplement: Supplementary file 1 — Supplementary Material 1 [file 12904_2024_1456_MOESM1_ESM.doc]

**Knowledge, attitudes and practices of critical care unit personnel regarding pediatric palliative care: A cross-sectional study**

**Basic Information**

| 1.Gender |
| --- |
| 1. Male |
| b. Female |
| 2. Age |
| a.<30 years-old |
| b.≥30 years-old |
| 3. PICU pation |
| a.Physician |
| b.Nurse |
| 4. Education level |
| a.Bachelor’s degree or below |
| b.Master’s degree or above |
| 5. Years of work experience |
| a.≤5 years |
| b.5–10 years |
| c.11–15 years |
| d.≥16 years |
| 6. Region |
| a.East China (Shanghai and Suzhou) |
| b.Southwest China (Sichuan-Chongqing, Yunnan) |
| 7. Previous training in pediatric palliative care |
| a.Yes |
| b.No |
| 8. Type of training received |
| a.Theory |
| b.Technology |
| c.Team formation and management |
| 9. Availability of pediatric palliative care in the department |
| a.Available |
| b.Not available |

**Knowledge Assessment**

| K1. Pediatric palliative care involves the physical, psychological and spiritual care of children with life-threatening diseases as well as the support of their families so as to provide an optimal quality of life for the children and their families | a.True | b.False | c.Unclear |
| --- | --- | --- | --- |
| K2. Adult palliative care is performed mainly for adults with tumors, whereas pediatric palliative care is also performed for children with non-malignant life-limiting conditions that encompass a broader spectrum of diseases | a.True | b.False | c.Unclear |
| L3. Pediatric palliative care is used only in patients with potentially treatable life-threatening diseases after treatment has failed, such as tumors and irreversible failure of the heart, liver or kidneys (false) | a.True | b.False | c.Unclear |
| K4. Pediatric palliative care includes the control of pain and other symptom, comfort care, and support for parents | a.True | b.False | c.Unclear |
| K5. Strong opioids should be considered in children who are more sensitive to pain than adults and when the cause of pain is difficult to identify (false) | a.True | b.False | c.Unclear |
| K6. In addition to the assessment of vital signs, pain scales should be used to assess pain | a.True | b.False | c.Unclear |
| K7. Opioid substitution therapy to reduce iatrogenic withdrawal syndrome should be considered regardless of the previous drug dose, duration of treatment or drug used (false) | a.True | b.False | c.Unclear |
| K8. Comfort care mainly involves the creation of an environment that is comfortable and safe for the child, reducing discomfort, and not performing unnecessary examinations or treatments | a.True | b.False | c.Unclear |
| K9. The presence of parents or caregivers during routine care and intervention provides comfort for the child, reduces parental stress and anxiety levels, and improves care satisfaction | a.True | b.False | c.Unclear |
| K10. Sleep deprivation is an important stressor for critically ill survivors, and improvements in environmental noise and light levels should be considered | a.True | b.False | c.Unclear |
| K11. Pediatric palliative care should be incorporated into the routine care of patients with life-limiting or life-threatening conditions from the time of diagnosis | a.True | b.False | c.Unclear |
| K12. Pediatric palliative care allows families in need to get help from interdisciplinary teams to improve the quality of life during treatment rather than give up treatment | a.True | b.False | c.Unclear |
| K13. Active treatment of the primary disease should have different focuses at different stages of the disease and should not aim to prolong survival time at the expense of quality of life | a.True | b.False | c.Unclear |

**Attitude Assessment**

| A1: Pediatric palliative care should be implemented to optimize quality of life if invasive therapy would cause discomfort and have little effect on the underlying disease (positive) | a.Extremely positive | b.Positive | c. Neutral | d.Negative | e. Extremely negative |
| --- | --- | --- | --- | --- | --- |
| A2. Pediatric palliative care can improve outcomes for children and their families (positive) | a.Extremely positive | b.Positive | c. Neutral | d.Negative | e. Extremely negative |
| A3. Pediatric palliative care can make the family lose hope (negative) | a.Extremely positive | b.Positive | c. Neutral | d.Negative | e. Extremely negative |
| A4. Pediatric palliative care will result in the family being placed under pressure in a variety of ways (negative) | a.Extremely positive | b.Positive | c. Neutral | d.Negative | e. Extremely negative |
| A5. Pediatric palliative care should be implemented by a multidisciplinary team composed of doctors, nurses, social workers, pharmacists, physiotherapists and others (positive) | a.Extremely positive | b.Positive | c. Neutral | d.Negative | e. Extremely negative |
| A6. Pediatric palliative care should be integrated into the intensive care unit and jointly provided by the Department of Critical Care Medicine and palliative care team (positive) | a.Extremely positive | b.Positive | c. Neutral | d.Negative | e. Extremely negative |
| A7. Nurses, educators and researchers should do more to provide guidance for the application and evaluation of high-quality pediatric palliative care (positive) | a.Extremely positive | b.Positive | c. Neutral | d.Negative | e. Extremely negative |
| Which of the following factors influence pediatric palliative care: | | | | | |
| A8. political-economic factors | | | | | |
| A8.1 insufficient human resources and inadequate structural organization | a.Pretty much | b.Much | c.General | d.Little | e.Pretty little |
| A8.2 insufficient financial resources and drug availability | a.Pretty much | b.Much | c.General | d.Little | e.Pretty little |
| A8.3 level of medical insurance reimbursement | a.Pretty much | b.Much | c.General | d.Little | e.Pretty little |
| A9. medical personnel-related factors (negative) | | | | | |
| A9.1 Time pressures | a.Pretty much | b.Much | c.General | d.Little | e.Pretty little |
| A9.2 lack of education/training/knowledge (communication skills, pain assessment and management) | a.Pretty much | b.Much | c.General | d.Little | e.Pretty little |
| A9.3 emotional distress/discomfort/sadness | a.Pretty much | b.Much | c.General | d.Little | e.Pretty little |
| A10. family-related factors (negative) | | | | | |
| A10.1 misunderstanding of the role of palliative care and fear of being abandoned by medical personnel | a.Pretty much | b.Much | c.General | d.Little | e.Pretty little |
| A10.2 misunderstanding of the prognosis or goals of treatment | a.Pretty much | b.Much | c.General | d.Little | e.Pretty little |
| A11. social factors (negative) | | | | | |
| A11.1 views that promote life and are not compatible with treatment withdrawal | a.Pretty much | b.Much | c.General | d.Little | e.Pretty little |
| A11.2 misunderstanding of pediatric palliative care (the role of the hospice, voluntary abandonment) | a.Pretty much | b.Much | c.General | d.Little | e.Pretty little |
| A11.3 treatment/cure-oriented views | a.Pretty much | b.Much | c.General | d.Little | e.Pretty little |
| A12. clinical implementation and standardization-related factors (negative) | | | | | |
| A12.1 timing of the initiation of palliative care and uncertainty about the prognosis | a.Pretty much | b.Much | c.General | d.Little | e.Pretty little |
| A12.2 lack of specific reference guidelines | a.Pretty much | b.Much | c.General | d.Little | e.Pretty little |
| A12.3 lack of specific implementation standards (team formation, bereavement care, etc.) | a.Pretty much | b.Much | c.General | d.Little | e.Pretty little |

**Practice Assessment**

| How frequently do you (and your team) implement the following if necessary. | | | | | |
| --- | --- | --- | --- | --- | --- |
| P1: analgesia (positive) | a.Always | b.Often | c.Sometimes | d.Rarely | e.Never |
| P2: sedation (positive) | a.Always | b.Often | c.Sometimes | d.Rarely | e.Never |
| P3. application of neuromuscular blockers (positive) | a.Always | b.Often | c.Sometimes | d.Rarely | e.Never |
| P4: screening and prevention of delirium (e.g., minimizing overall sedative exposure) (positive) | a.Always | b.Often | c.Sometimes | d.Rarely | e.Never |
| P5: assessment, prevention and mitigation of iatrogenic withdrawal syndrome (positive) | a.Always | b.Often | c.Sometimes | d.Rarely | e.Never |
| P6: environment optimization (positive); P7: basic symptom management (positive) | a.Always | b.Often | c.Sometimes | d.Rarely | e.Never |
| P7: basic symptom management (positive) | a.Always | b.Often | c.Sometimes | d.Rarely | e.Never |
| P8: high-quality communication (positive) | a.Always | b.Often | c.Sometimes | d.Rarely | e.Never |
| P9: end-of-life care (positive) | | | | | |
| P9.1: withdrawal of life support equipment | a.Always | b.Often | c.Sometimes | d.Rarely | e.Never |
| P9.2: support for parents | a.Always | b.Often | c.Sometimes | d.Rarely | e.Never |
