# Supplementary material for: Knowledge, attitudes and practices of critical care unit personnel regarding pediatric palliative care: a cross-sectional study
Source: BMC Palliat Care. 2024 May 21;23:125. doi: 10.1186/s12904-024-01456-w (PMC11106871; doi:10.1186/s12904-024-01456-w)
Supplement: Supplementary file 2 — Supplementary Material 2 [file 12904_2024_1456_MOESM2_ESM.docx]

# Supplementary Table S1. Knowledge, attitude and practice scores stratified according to the baseline characteristics of the study participants.

| Characteristic | *n* (%) | Knowledge score | | Attitude score | | Practice score | |
| --- | --- | --- | --- | --- | --- | --- | --- |
|  |  | Mean ± SD | *P* | Mean ± SD | *P* | Mean ± SD | *P* |
| Total scores | 204 (100%) | 9.75 ± 2.90 |  | 38.30 ± 3.80 |  | 35.48 ± 5.72 |  |
| Gender |  |  | 0.727 |  | 0.473 |  | 0.130 |
| Male | 29 (14.22%) | 9.72 ± 2.71 |  | 37.83 ± 4.62 |  | 34.07 ± 5.44 |  |
| Female | 175 (85.78%) | 9.75 ± 2.94 |  | 38.38 ± 3.65 |  | 35.71 ± 5.74 |  |
| Age |  |  | 0.095 |  | 0.547 |  | 0.161 |
| <30 years-old | 93 (45.59%) | 9.52 ± 2.87 |  | 38.12 ± 4.19 |  | 34.86 ± 6.54 |  |
| ≥30 years-old | 111 (54.41%) | 9.95 ± 2.93 |  | 38.44 ± 3.45 |  | 36.01 ± 4.90 |  |
| PICU pation |  |  | <0.001 |  | 0.111 |  | 0.287 |
| Physician | 46 (22.55%) | 10.87 ± 2.01 |  | 39.11 ± 3.78 |  | 36.23 ± 4.19 |  |
| Nurse | 158 (77.45%) | 9.42 ± 3.04 |  | 38.06 ± 3.78 |  | 35.26 ± 6.08 |  |
| Education level |  |  | 0.0244 |  | 0.081 |  | 0.100 |
| Bachelor’s degree or below | 166 (81.37%) | 9.60 ± 2.90 |  | 38.08 ± 3.82 |  | 35.18 ± 5.94 |  |
| Master’s degree or above | 38 (18.63%) | 10.42 ± 2.85 |  | 39.27 ± 3.61 |  | 36.80 ± 4.47 |  |
| Years of work experience |  |  | 0.434 |  | 0.323 |  | 0.233 |
| ≤5 years | 70 (34.31%) | 9.53 ± 3.04 |  | 38.45 ± 4.15 |  | 34.69 ± 6.04 |  |
| 6–10 years | 66 (32.35%) | 9.82 ± 2.91 |  | 37.89 ± 3.33 |  | 35.58 ± 6.09 |  |
| 11–15 years | 40 (19.61%) | 9.63 ± 2.79 |  | 37.95 ± 3.74 |  | 36.99 ± 5.43 |  |
| ≥16 years | 28 (13.73%) | 10.32 ± 2.74 |  | 39.38 ± 3.97 |  | 35.05 ± 3.93 |  |
| Region |  |  | 0.834 |  | 0.118 |  | 0.976 |
| East China (Shanghai and Suzhou) | 93 (45.59%) | 9.87 ± 2.70 |  | 37.84 ± 4.14 |  | 35.58 ± 5.68 |  |
| Southwest China (Sichuan-Chongqing, Yunnan) | 111 (54.41%) | 9.65 ± 3.07 |  | 38.68 ± 3.46 |  | 35.40 ± 5.77 |  |
| Previous training in pediatric palliative care |  |  | 0.005 |  | 0.271 |  | 0.586 |
| Yes | 57 (27.94%) | 10.53 ± 2.43 |  | 38.77 ± 3.99 |  | 35.47 ± 5.79 |  |
| No | 147 (72.06%) | 9.45 ± 3.02 |  | 38.11 ± 3.72 |  | 35.48 ± 5.71 |  |
| Type of training received |  |  |  |  |  |  |  |
| Theory | 53 (25.98%) | 10.66 ± 2.25 | 0.285 | 38.83 ± 4.02 | 0.236 | 35.77 ± 5.48 | 0.133 |
| Technology | 10 (4.90%) | 10.70 ± 2.71 | 0.447 | 41.65 ± 3.25 | 0.004 | 34.95 ± 6.15 | 0.833 |
| Team formation and management | 14 (6.86%) | 10.64 ± 2.53 | 0.784 | 40.06 ± 4.15 | 0.072 | 36.00 ± 7.26 | 0.669 |
| Availability of pediatric palliative care in the department |  |  | 0.065 |  | 0.409 |  | 0.429 |
| Available | 97 (47.55%) | 10.26 ± 2.29 |  | 38.53 ± 3.92 |  | 35.31 ± 5.93 |  |
| Not available | 107 (52.45%) | 9.29 ± 3.31 |  | 38.09 ± 3.69 |  | 35.63 ± 5.54 |  |

SD: standard deviation.

**Supplementary Table S2. Responses to the questions in the knowledge dimension.**

| Item | *n* (%) | |
| --- | --- | --- |
|  | True | False/unclear |
| K1. Pediatric palliative care involves the physical, psychological and spiritual care of children with life-threatening diseases as well as the support of their families so as to provide an optimal quality of life for the children and their families | 176 (86.27%) | 28 (13.73%) |
| K2. Adult palliative care is performed mainly for adults with tumors, whereas pediatric palliative care is also performed for children with non-malignant life-limiting conditions that encompass a broader spectrum of diseases | 144 (70.59%) | 60 (29.41%) |
| L3. Pediatric palliative care is used only in patients with potentially treatable life-threatening diseases after treatment has failed, such as tumors and irreversible failure of the heart, liver or kidneys (false) | 78 (38.24%) | 126 (61.76%) |
| K4. Pediatric palliative care includes the control of pain and other symptom, comfort care, and support for parents | 184 (90.20%) | 20 (9.80%) |
| K5. Strong opioids should be considered in children who are more sensitive to pain than adults and when the cause of pain is difficult to identify (false) | 119 (58.33%) | 85 (41.67%) |
| K6. In addition to the assessment of vital signs, pain scales should be used to assess pain | 193 (94.61%) | 11 (5.39%) |
| K7. Opioid substitution therapy to reduce iatrogenic withdrawal syndrome should be considered regardless of the previous drug dose, duration of treatment or drug used (false) | 95 (46.57%) | 109 (53.43%) |
| K8. Comfort care mainly involves the creation of an environment that is comfortable and safe for the child, reducing discomfort, and not performing unnecessary examinations or treatments | 140 (68.63%) | 64 (31.37%) |
| K9. The presence of parents or caregivers during routine care and intervention provides comfort for the child, reduces parental stress and anxiety levels, and improves care satisfaction | 178 (87.25%) | 26 (12.75%) |
| K10. Sleep deprivation is an important stressor for critically ill survivors, and improvements in environmental noise and light levels should be considered | 187 (91.67%) | 17 (8.33%) |
| K11. Pediatric palliative care should be incorporated into the routine care of patients with life-limiting or life-threatening conditions from the time of diagnosis | 177 (86.76%) | 27 (13.24%) |
| K12. Pediatric palliative care allows families in need to get help from interdisciplinary teams to improve the quality of life during treatment rather than give up treatment | 174 (85.29%) | 30 (14.71%) |
| K13. Active treatment of the primary disease should have different focuses at different stages of the disease and should not aim to prolong survival time at the expense of quality of life | 144 (70.59%) | 60 (29.41%) |

**Supplementary Table S3. Subgroup analyses**

| Dimension/item | PICU occupation | | *P* | Previous training | | *P* |
| --- | --- | --- | --- | --- | --- | --- |
|  | Physician | Nurse |  | Trained | Untrained |  |
| Knowledge total score | 10.87 ± 2.08 | 9.42 ± 3.04 | <0.001 | 10.53 ± 2.43 | 9.45 ± 3.02 | 0.017 |
| Attitude |  |  |  |  |  |  |
| Considerations for selection of pediatric palliative care (A1–A4) | 15.98 ± 2.43 | 15.03 ± 2.56 | 0.026 | 15.58 ± 2.51 | 15.11 ± 2.57 | 0.239 |
| Composition of the pediatric palliative care team (A5–A7) | 13.22 ± 1.53 | 12.58 ± 2.02 | 0.023 | 12.82 ± 1.81 | 12.68 ± 1.98 | 0.634 |
| Political-economic factors (A8.1–A8.3) | 5.41 ± 2.07 | 5.77 ± 2.29 | 0.350 | 5.82 ± 2.25 | 5.63 ± 2.25 | 0.585 |
| Medical staffs-related factors (A9.1–A9.3) | 6.30 ± 2.04 | 6.32 ± 2.06 | 0.972 | 6.16 ± 2.08 | 6.37 ± 2.05 | 0.501 |
| Family-related factors (A10.1–A10.2) | 4.15 ± 1.81 | 4.27 ± 1.60 | 0.665 | 4.23 ± 1.71 | 4.25 ± 1.63 | 0.927 |
| Social factors (A11.1–A11.3) | 6.11 ± 2.06 | 6.60 ± 2.41 | 0.210 | 6.60 ± 2.53 | 6.45 ± 2.27 | 0.687 |
| Clinical implementation and guideline-related factors (A12.1–A12.3) | 5.67 ± 2.07 | 6.29 ± 2.28 | 0.094 | 6.18 ± 2.30 | 6.14 ± 2.17 | 0.925 |
| Practice |  |  |  |  |  |  |
| Analgesia (P1) | 4.37 ± 0.68 | 4.22 ± 0.83 | 0.272 | 4.32 ± 0.76 | 4.23 ± 0.82 | 0.501 |
| Sedation (P2) | 4.00 ± 0.84 | 4.03 ± 0.84 | 0.858 | 4.11 ± 0.75 | 3.99 ± 0.88 | 0.367 |
| Application of neuromuscular blockers (P3) | 3.04 ± 1.03 | 3.72 ± 0.88 | <0.001 | 3.47 ± 0.95 | 3.60 ± 0.96 | 0.404 |
| Screening for/preventing delirium (e.g., minimizing sedative exposure) (P4) | 4.07 ± 0.85 | 3.89 ± 0.85 | 0.226 | 3.93 ± 0.80 | 3.93 ± 0.87 | 0.987 |
| Assessing and preventing iatrogenic withdrawal syndrome (P5) | 4.11 ± 0.71 | 3.94 ± 0.82 | 0.214 | 3.91 ± 0.79 | 4.01 ± 0.80 | 0.447 |
| Environment optimization (P6) | 4.15 ± 0.76 | 4.01 ± 0.81 | 0.300 | 4.09 ± 0.74 | 4.03 ± 0.83 | 0.630 |
| Basic symptom management (P7) | 4.15 ± 0.67 | 3.89 ± 0.84 | 0.049 | 3.89 ± 0.77 | 3.97 ± 0.82 | 0.573 |
| High-quality communication (P8) | 4.39 ± 0.68 | 4.15 ± 0.82 | 0.065 | 4.23 ± 0.76 | 4.19 ± 0.81 | 0.763 |
| End-of-life care (P9) | 7.89 ± 1.90 | 6.84 ± 1.85 | <0.001 | 7.05 ± 1.77 | 7.09 ± 1.89 | 0.902 |

Supplementary Table S4 The results of Model fit.

| Indicators | Reference | Results |
| --- | --- | --- |
| RMSEA | <0.08 Good | 0.065 |
| SRMR | <0.08 Good | 0.073 |
| TLI | >0.8 Good | 0.846 |
| CFI | >0.8 Good | 0.857 |

Supplementary Table S5 The results of SEM.


| Model paths |  | Total effects | | Direct Effect | | Indirect effect | |
| --- | --- | --- | --- | --- | --- | --- | --- |
|  |  | β (95% CI) | P | β (95% CI) | P | β (95% CI) | P |
| Asum <- |  |  |  |  |  |  |  |
|  | Ksum | 0.69(0.28,1.10) | 0.001 | 0.69(0.28,1.10) | 0.001 |  |  |
| Psum <- |  |  |  |  |  |  |  |
|  | Asum | 1.18(0.81,1.56) | <0.001 | 1.18(0.81,1.56) | <0.001 |  |  |
|  | Ksum | 0.54(0.08,1.01) | 0.021 | -0.27(-0.64,0.09) | 0.144 | 0.82(0.36,1.28) | <0.001 |
